# Neuroendocrine neoplasm of the gallbladder: Clinical features, surgical efficacy, and prognosis

**DOI:** 10.1002/cam4.5846

**Published:** 2023-03-23

**Authors:** Xin Wu, Binglu Li, Tao Hong, Wei Liu, Xiaodong He, Chaoji Zheng

**Affiliations:** ^1^ Department of General Surgery Peking Union Medical College Hospital, Chinese Academy of Medical Sciences & Peking Union Medical College Beijing China

**Keywords:** adenocarcinoma, gallbladder, neuroendocrine neoplasm, prognosis, surgery

## Abstract

**Background:**

Neuroendocrine neoplasm (NEN) of the gallbladder is rare. It is usually asymptomatic and occurs in older adults. Its clinicopathological characteristics remain controversial, and the diagnosis and treatment strategies are usually based on models of adenocarcinoma. The present study aimed to investigate the clinical characteristics and prognosis of gallbladder NEN.

**Methods:**

The data of patients with gallbladder NEN admitted to Peking Union Medical College Hospital was reviewed, and a database was established for retrospective analysis. Clinicopathological features were analyzed descriptively and the prognosis was studied according to different factors. The Kaplan–Meier curve was used to describe the cumulative survival rate.

**Results:**

In total, 22 patients with gallbladder NEN were included in this study. There were 10 male (45.5%) and 12 female (54.5%) patients with a median age of onset of NEN at 57.5 (49.0, 62.3) years. Abdominal discomfort was the most common symptom. Twenty patients (90.9%) underwent surgery, and two patients (9.1%) with unresectable lesions underwent a biopsy. Twenty‐one patients were followed up. The 1‐, 2‐, and 3‐year cumulative overall survival rates of all patients and patients with resectable lesions were 65.9%, 54.9%, and 48.1%, and 72.9%, 60.7%, and 53.1%, respectively. Patients with resectable lesions had a better cumulative overall survival rate than those who with unresectable lesions (*p* < 0.001).

**Conclusion:**

Gallbladder NEN is more common in the elderly and has a slight female predominance. The most common symptom is abdominal discomfort. Surgery is the first choice of treatment for this rare disease. The prognosis of gallbladder NEN is generally poor. Patients with resectable lesions have a better prognosis.

## Graphical abstract

We found that gallbladder NEN patients with resectable lesions had a better prognosis. The 1‐, 2‐, and 3‐year cumulative OS rates of all patients and patients with resectable lesions were 65.9%, 54.9%, and 48.1%, and 72.9%, 60.7%, and 53.1%, respectively. But patients with different age, sex, tumor size and tumor nature had no different cumulative OS rates.

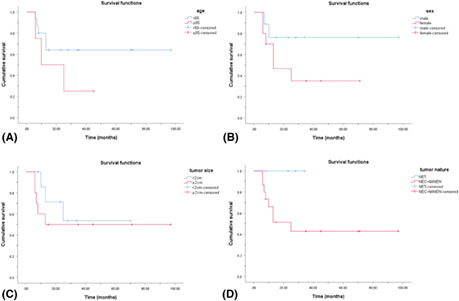

## 1 INTRODUCTION

Neuroendocrine neoplasm (NEN) is a rare malignant disease with indolent clinical features.^1^ Its incidence increased from 1.09 per 100,000 people in 1973 to 6.98 per 100,000 people in 2012, much faster than the rate of all malignant neoplasms in the same period.^1^ Approximately two‐thirds of NENs occur in the digestive system, mainly in the rectum, pancreas, stomach, and ileum.^2^, ^3^ NENs are classified as mixed endocrine non‐endocrine neoplasm (MiNEN), neuroendocrine carcinoma (NEC), and neuroendocrine tumor (NET) according to the tumor composition and degree of differentiation in the latest edition of the WHO classification.^4^ Gallbladder cancer accounts for approximately 1% of all cancers worldwide, with the most common subtype being gallbladder adenocarcinoma.^5^, ^6^ Gallbladder NEN is extremely rare, accounting for 2% of all gallbladder carcinomas and 0.5% of all NENs.^3^, ^7^, ^8^, ^9^ Some NENs of the digestive system, such as insulinomas and gastrinomas, can be detected at an early stage because of their symptomatic features. However, gallbladder NEN is usually asymptomatic and diagnosed at an advanced stage.^10^

Some previous studies suggested that gallbladder NEN was more common in the elderly and females, and its most common subtype was NEC.^9^, ^11^, ^12^, ^13^, ^14^ However, the clinical characteristics of gallbladder NEN remain controversial, and there is still no standard diagnosis and treatment strategy due to its low incidence. To date, the vast majority of treatment and research on gallbladder NEN has been based on a model of gallbladder adenocarcinoma. Gallbladder NEN was reported to have similar general features and worse prognosis compared with adenocarcinoma.^10^, ^15^ Most studies of gallbladder NEN are case reports, and even case series reports are few. In order to better understand this rare disease, more studies about gallbladder NEN should be encouraged. We aimed to investigate the clinical characteristics and prognosis of gallbladder NEN by retrospective analysis of cases from a large tertiary hospital, to increase our understanding of this rare disease.

## 2 MATERIALS AND METHODS

### 2.1 Patients

Data of 11,260 patients who underwent gallbladder surgery and 2844 patients with NEN treated at our hospital between January 2012 and December 2021 were obtained from the electronic medical record system of Peking Union Medical College Hospital. Patients who met the following criteria were included in the study: (1) patients with gallbladder lesion that was diagnosed by pre‐operative imaging and intra‐operative examination; (2) who underwent cholecystectomy or lesion biopsy, with or without lymph node dissection, liver resection, or combined organ resection; (3) in whom gallbladder NEN was confirmed by paraffin pathology; (4) whose medical records were complete and available. Patients with a pathological diagnosis of other gallbladder carcinomas and those with incomplete medical records were excluded. A database was established and used for the analysis. The demographic characteristics (sex, age, body mass index), clinical symptoms, smoking and drinking history, underlying diseases (hypertension, diabetes, coronary heart disease, cholelithiasis), carcinoembryonic antigen and carbohydrate antigen 19‐9 test levels, surgical details, pathological information, and prognosis were studied and analyzed.

This study was reviewed and approved by the institutional review board of Peking Union Medical College Hospital (I‐22PJ118). The requirement of informed consent for the publication of data was waived owing to the retrospective nature.

### 2.2 Classification and definition

NEN was defined and classified according to the 2019 WHO classification of tumors of the digestive system (5th edition).^4^ Grades 1, 2, and 3 were defined according to the following criteria: G1, Ki‐67 index <3%, mitotic rate <2 per 2 mm^2^; G2, Ki‐67 index of 3%–20%, mitotic rate of 2–20 per 2 mm^2^; G3, Ki‐67 index >20%, mitotic rate >20 per 2 mm^2^. The American Joint Committee on Cancer staging manual (8th edition) was used for TNM staging.^16^ Post‐operative complications were defined and classified according to the Clavien–Dindo system.^17^ The upper limits of the reference values for carcinoembryonic antigen and carbohydrate antigen 19‐9 in the present study were 5 ng/mL and 34 U/mL, respectively.

### 2.3 Statistical analysis

All analyses were performed using Statistical Package for Social Sciences software (version 25.0; IBM). Categorical variables were presented as absolute numbers and percentages. Continuous variables were presented as median (25th, 75th). The cumulative survival rates were described by Kaplan–Meier curves with the log‐rank test. Statistical significance was set at *p* < 0.05.

## 3 RESULTS

**FIGURE 1:**
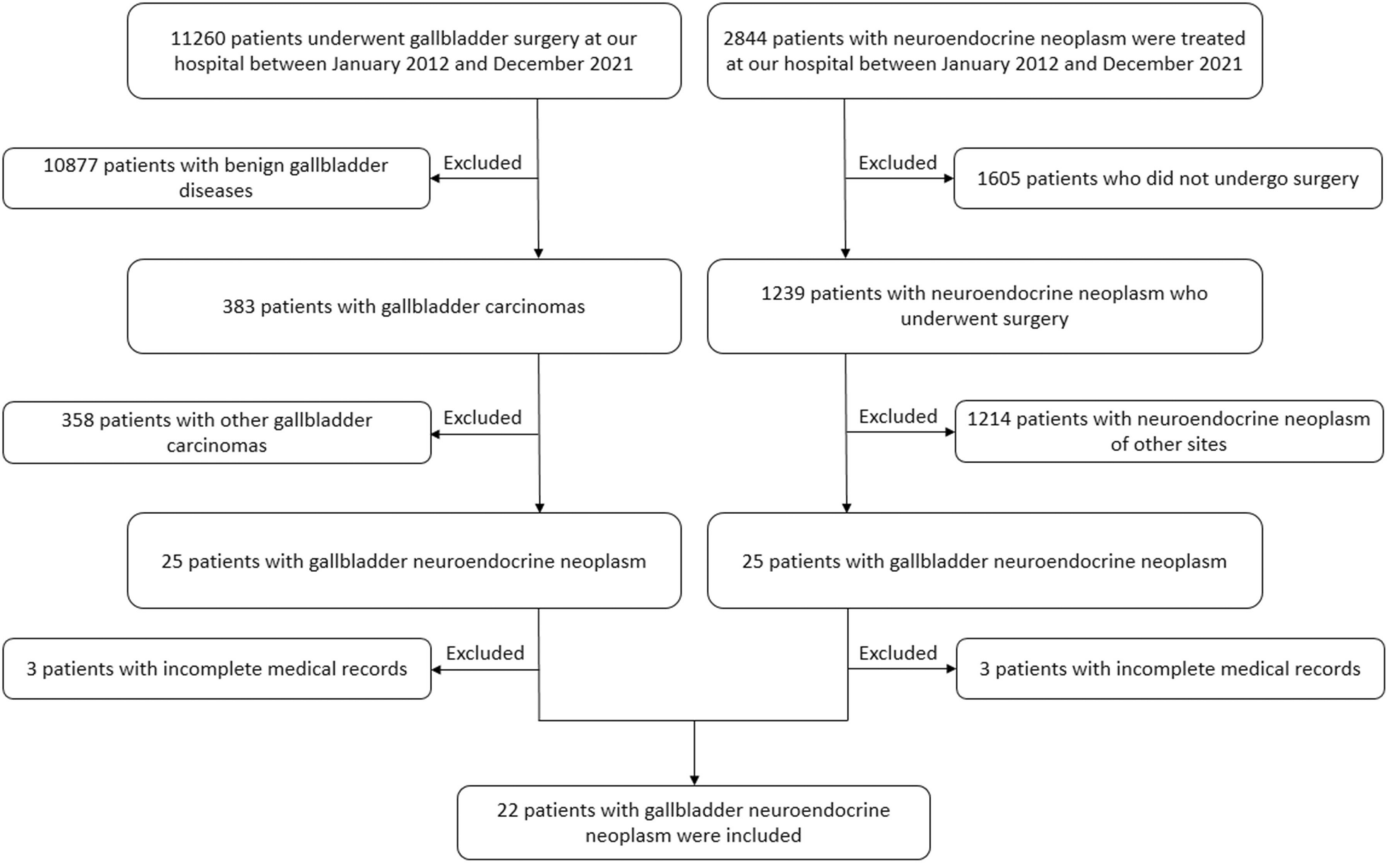
The flow diagram of the study participant selection. Two different screening paths resulted in the same enrolled patients.

In total, 11,260 patients underwent gallbladder surgery at our hospital between January 2012 and December 2021. Twenty‐two patients with gallbladder NEN were included in this study based on the inclusion and exclusion criteria. The medical records of 2844 patients with NEN in the same period were also reviewed to ensure the accuracy of the number of enrolled patients. The flow diagram of the study participant selection is shown in Figure 1. The general information of all 22 patients is presented in Table 1. Twenty patients (90.9%) underwent surgery and two patients (9.1%) with unresectable lesions only underwent biopsy.

**TABLE 1:** General information of 22 patients with gallbladder neuroendocrine neoplasm.

Variables	Value
Sex male/female (*n*)	10/12
Age (years)	57.5 (49.0, 62.3) (range 28–79)
BMI (kg/m^2^)	22.9 (21.5, 24.7) (range 17.4–31.2)
Abdominal discomfort (*n*)	13
Nausea and vomiting (*n*)	4
Weight loss (*n*)	4
Fever (*n*)	2
Jaundice (*n*)	1
Smoking (*n*)	6
Drinking (*n*)	3
Hypertension (*n*)	8
Diabetes (*n*)	3
Coronary heart disease (*n*)	2
Cholelithiasis (*n*)	6
CEA > 5 ng/mL (*n*)	4
CA19‐9 > 34 U/mL (*n*)	1

Abbreviations: BMI, body mass index; CA19‐9, carbohydrate antigen 19‐9; CEA, carcinoembryonic antigen.

Of the 20 patients who underwent surgery, all underwent cholecystectomy, 13 underwent lymph node dissection, and nine underwent liver resection. The range of lymph node dissection included groups 8, 12, and 13. Liver resection method included wedge liver and segment 4/5 resections. Surgical and pathological information of the 20 patients who underwent surgery is shown in Table 2. All 4 patients with NET were highly differentiated, low grade, and had a Ki‐67 index of 1%. They were classified as G1. Ten patients with NEC were poorly differentiated and high grade. For six patients with MiNEN, the neuroendocrine components were all poorly differentiated, and the adenocarcinoma components were poorly differentiated in 5 patients and highly differentiated in 1 patient. The immunohistochemical staining results of the 20 patients who underwent surgery are shown in Table 3. Three patients experienced postoperative complications, such as vomiting (*n* = 1), abdominal infection (*n* = 1), and bile duct infection (*n* = 1). All of them recovered after conservative treatment. According to the Clavien–Dindo system, one and two patients were classified as grade I and II, respectively.

**TABLE 2:** Surgical and pathological information of the 20 patients with gallbladder neuroendocrine neoplasm who underwent surgery.

Variables	Value
Laparoscopic/open surgery (*n*)	15/5
ASA I/II (*n*)	2/18
Surgery time (min)	162.5 (101.3, 200.0) (range 40–255)
Surgery bleeding (mL)	100 (30, 300) (range 30–600)
R0/R1 resection (*n*)	19/1
Tumor size (cm)	1.8 (1.0, 2.5) (range 0.4–6.5)
NET/NEC/MiNEN (*n*)	4/10/6
Ki‐67 index (%)	70 (33, 80) (range 1–90)
No. of dissected lymph nodes (*n*)	7 (4, 13) (range 1–20)
No. of positive lymph nodes (*n*)	1 (0, 2) (range 0–20)
TNM (*n*)	
I	5
II	3
IIIA	4
IIIB	6
IVB	2

Abbreviations: ASA, American Society of Anesthesiologists; MiNEN, mixed endocrine non‐endocrine neoplasm; NEC, neuroendocrine carcinoma; NET, neuroendocrine tumor.

**TABLE 3:** The immunohistochemical stain results of the 20 patients with gallbladder neuroendocrine neoplasm who underwent surgery.

Variables	No. of test (*n*)	No. of positive (*n*)	%
CgA	20	17	85.0
Syn	19	18	94.7
CD56	13	13	100
CK19	9	7	77.8

Seventeen patients with tumor stage T2 and above, or positive lymph nodes, were recommended to undergo adjuvant treatment. Nine of them accepted, but the other eight patients refused adjuvant treatment due to their poor general conditions. Chemotherapy was performed in eight patients. The treatment regimens were oxaliplatin combined with capecitabine in five and carboplatin combined with etoposide in three. Two patients received radiotherapy in addition to chemotherapy. Targeted therapy was performed in one patient with the protocol of lenvatinib.

**FIGURE 2:**
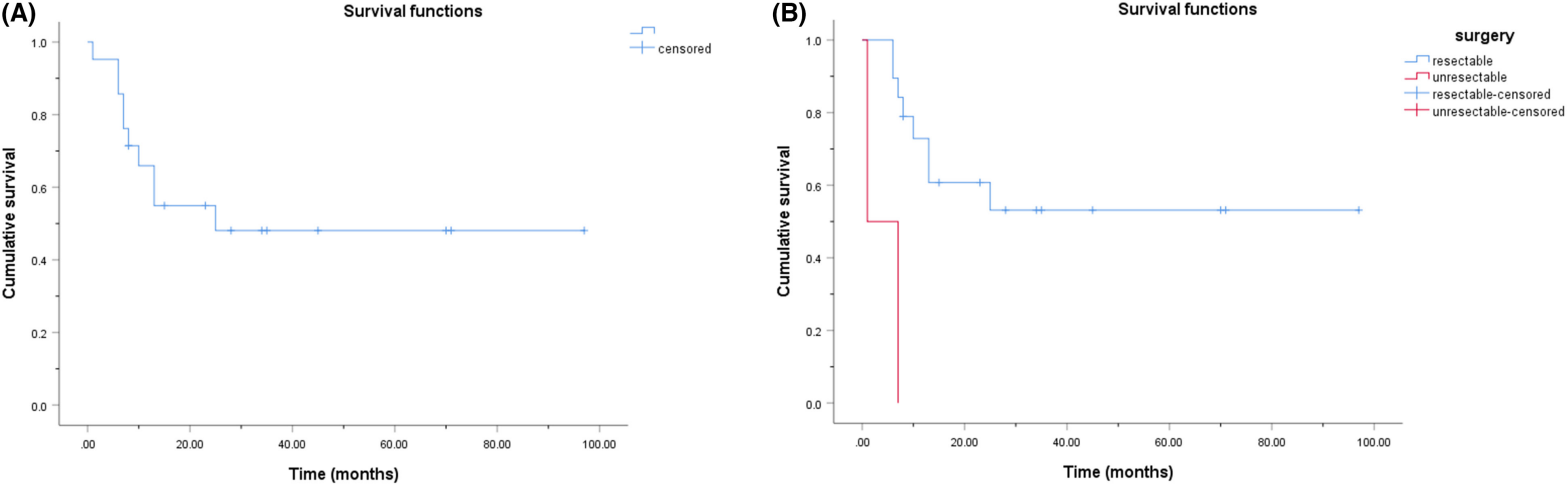
Kaplan–Meier cumulative OS curves for patients with gallbladder NEN. (A) The 1‐, 2‐, and 3‐year cumulative OS rates of all 21 patients with follow‐up information were 65.9%, 54.9%, and 48.1%, respectively. (B) The 1‐, 2‐, and 3‐year cumulative OS rates of the 19 patients with resectable lesions were 72.9%, 60.7%, and 53.1%, respectively. Patients with resectable lesions had a significantly better cumulative OS rate than those with unresectable lesions (*p* < 0.001).

**FIGURE 3:**
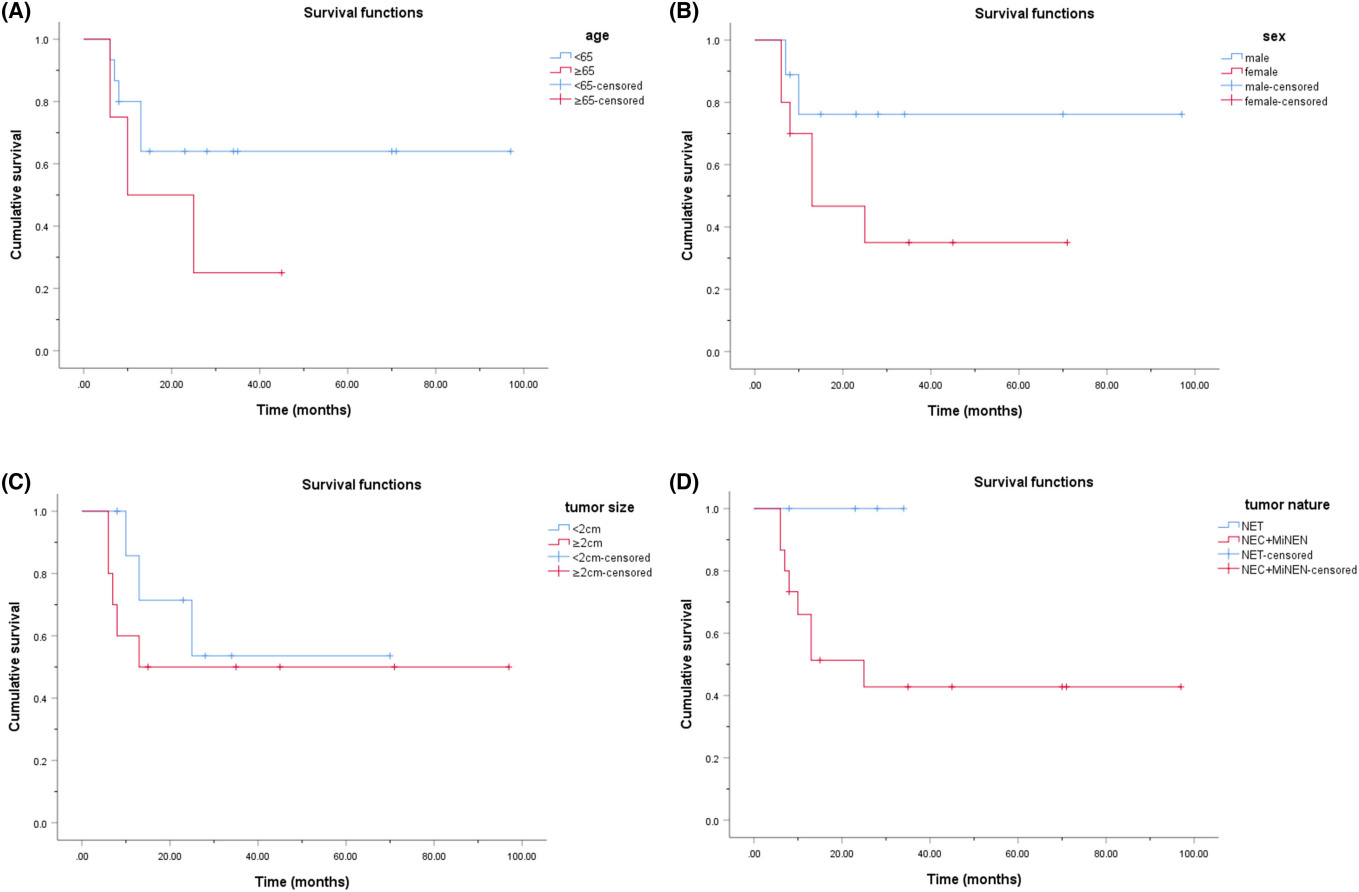
Kaplan–Meier cumulative OS curves for the 19 gallbladder NEN patients with resectable lesions according to (A) age (*p* = 0.206), (B) sex (*p* = 0.146), (C) tumor size (*p* = 0.437), and (D) tumor nature (*p* = 0.109). No significant difference was found between different groups.

As of April 2022, 19 of the 20 patients who underwent surgery were followed up at a median time of 15 (8, 35) months. One patient was lost to follow‐up. Two patients with unresectable lesions died at 1 and 7 months after the biopsy, respectively. A total of 21 patients had follow‐up information. The cumulative overall survival (OS) rates were described using Kaplan–Meier curves. The 1‐, 2‐, and 3‐year cumulative OS rates of all 21 patients were 65.9%, 54.9%, and 48.1%, respectively (Figure 2A). Patients with resectable lesions had a better cumulative OS rate than those with unresectable lesions (*p* < 0.001) (Figure 2B). The 1‐, 2‐, and 3‐year cumulative OS rates of the patients who underwent surgery were 72.9%, 60.7%, and 53.1%, respectively. Meanwhile, patients who underwent surgery were grouped and compared according to age, sex, tumor size, and tumor nature, and the cumulative OS rates of patients in different groups had no significant difference (*p* > 0.05) (Figure 3A–D).

## 4 DISCUSSION

Although the first case was reported as early as 1929, studies on gallbladder NEN are still rare.^3^ The clinical characteristics of the disease remain unclear, and diagnosis and treatment strategies are still not systematic. The present study reviewed the data of patients with gallbladder NEN from a large tertiary hospital, revealed the clinical characteristics and prognosis of this rare disease, which was conducive to deepening the understanding of gallbladder NEN.

Gallbladder NEN mainly occurs in older adults with a reported age of onset at 58–63 years.^3^, ^9^, ^10^ The male‐to‐female ratio has been reported differently in the literature. Some studies have suggested that the ratios are close^3^, ^10^; whereas, others suggested that females are more commonly affected.^7^, ^9^ The present study reported a slightly younger age of onset than that reported in the literature and a slight female predominance. Similar to most of the previous studies, this study also found abdominal discomfort to be the most common symptom of gallbladder NEN.^3^, ^10^, ^18^, ^19^, ^20^, ^21^, ^22^ Atypia of the symptoms is related to the anatomical location and function of the gallbladder. Carcinoid syndrome of the gallbladder NEN is exceedingly rare.^23^

Although there are several reports on the use of medical therapy, surgery is the first choice of treatment for patients with NEN.^24^, ^25^ Patients who underwent lesion removal had better outcomes than those who did not.^26^ Do et al^3^ reported 21 patients with gallbladder NEN, nine of whom underwent surgery. There was a significant difference in the progression‐free survival of surgical and nonsurgical patients (211.0 vs. 61.5 days, *p* = 0.036). Cen et al^7^ studied 248 patients with gallbladder NEN from the surveillance, epidemiology, and end results (SEER) database. Using Kaplan–Meier curves to describe survival time, they found that patients who underwent gallbladder surgery had significantly better overall and cancer‐specific survival than those who did not (*p* < 0.001). The present study revealed that patients with resectable lesions had a better OS than those with unresectable lesions, which is consistent with the literature.

The prognosis of gallbladder NEN is controversial because of its low incidence. Kamboj et al^27^ reported 19 cases of gallbladder NEN, 14 of which were followed up, with a median survival time of 3 months. Ayabe et al^8^ analyzed 300 patients from a large national database in America and revealed that the median OS time was 25 months. Gogna et al^28^ studied 482 patients with gallbladder NEN from the SEER database and found that the mean survival time was 37.11 months. In addition to survival time, reports on survival rates vary widely. The 1‐, 2‐, and 3‐year survival rates of gallbladder NEN were 20%–64%, 10%–38.8%, and 0%–31.1%, respectively.^9^, ^10^, ^29^ This study reported better survival rates in patients with gallbladder NEN than those in previously reported studies. This may be related to a number of factors, such as patient volume, race, and follow‐up time. Patients with gallbladder NEN with different characteristics may have different prognoses. Zhou et al^30^ retrospectively analyzed 446 patients with biliary NEN from the SEER database and found that young patients and patients with NET or smaller tumor size had a significantly better prognosis. Another SEER database study also revealed that age, tumor stage, extension, and grade were associated with the outcomes.^28^ Adjuvant therapy has also been associated with improved OS rates.^9^ This study compared patients according to their age, sex, tumor size, and tumor nature; however, no significant difference in prognosis was found. This may be because of the limited number of patients.

Owing to its low incidence and controversial clinical features, gallbladder NEN is often compared with adenocarcinoma, and several imaging features can be used to differentiate between the two.^31^, ^32^, ^33^ However, different studies have arrived at different conclusions regarding the prognosis of gallbladder NEN. Three studies, involving 10, 15, and 28 patients with gallbladder NEN, reported a worse prognosis in patients with gallbladder NEN than those with adenocarcinoma.^10^, ^11^, ^29^ In another study of 21 patients, the outcomes were similar.^3^ However, another study including 754 patients with gallbladder NEN revealed that the prognosis of gallbladder NEN was better than that of adenocarcinoma.^8^ More studies are needed to clarify the prognostic relationship between gallbladder NEN and adenocarcinoma.

In conclusion, this retrospective study evaluated the clinical characteristics and prognosis of gallbladder NEN. Abdominal discomfort was the most common symptom. CgA, Syn, CD56, and CK19 showed strong diagnostic significance. NEC was more common than NET and MiNEN. Patients with resectable lesions had a better prognosis. The 1‐, 2‐, and 3‐year cumulative OS rates of all patients and patients with resectable lesions were 65.9%, 54.9%, and 48.1%, and 72.9%, 60.7%, and 53.1%, respectively.

This study had some limitations. First, the number of patients was limited because of the low incidence rate of gallbladder NEN. It might lead to selection bias and multivariate analysis could not be performed. Second, the analysis factors could not be designed and planned in advance because of their retrospective nature. Therefore, prospective multicenter studies are needed to further clarify the clinical characteristics of gallbladder NEN.

## AUTHOR CONTRIBUTIONS

**Xin Wu:** Conceptualization (lead); data curation (lead); methodology (lead); writing – original draft (lead). **Binglu Li:** Conceptualization (lead); data curation (lead); funding acquisition (lead); methodology (supporting); supervision (equal); writing – review and editing (equal). **Tao Hong:** Conceptualization (supporting); data curation (supporting); methodology (supporting); supervision (supporting); writing – original draft (supporting); writing – review and editing (supporting). **Wei Liu:** Conceptualization (supporting); data curation (supporting); methodology (supporting); supervision (supporting); writing – original draft (supporting); writing – review and editing (supporting). **Xiaodong He:** Conceptualization (supporting); methodology (supporting); supervision (supporting); writing – original draft (supporting); writing – review and editing (supporting). **Chaoji Zheng:** Conceptualization (supporting); data curation (supporting); methodology (supporting); supervision (supporting); writing – original draft (supporting); writing – review and editing (supporting).

## FUNDING INFORMATION

The Chinese Academy of Medical Sciences Innovation Fund for Medical Sciences (2022‐I2M‐C&T‐A‐004) and the National High Level Hospital Clinical Research Funding (2022‐PUMCH‐B‐005).

## CONFLICT OF INTEREST STATEMENT

No potential conflict of interest concerning this study was reported.

## ETHICS STATEMENT

The ethical approval for conducting this experiment was granted by the Peking Union Medical College Hospital's Institutional Review Board (I‐22PJ118). Due to the retrospective nature of the research, informed consent for data publishing was not required.

## Data Availability

There is no extra information available.
